# Commentary: The Interleukin-2 T Cell System: A New Cell Growth Model

**DOI:** 10.3389/fimmu.2015.00414

**Published:** 2015-08-10

**Authors:** Kendall Arthur Smith

**Affiliations:** ^1^Division of Immunology, Department of Medicine, Weill Medical College, Cornell University, New York, NY, USA

**Keywords:** interleukin-2, T cell clones, IL-2 receptors, the quantal theory of immunity

Having created unique and novel cellular and molecular reagents, we could approach questions that had perplexed investigators interested in cell growth for over 50 years, i.e., the basis for variable cell cycle transit times of individual cells among genetically homogeneous cell populations (1). Studies of all cell populations, both prokaryote and eukaryote, revealed that within a cell population, the cell cycle times of individual cells follow a normal distribution when examined as a function of the division rate (the rate-normal distribution). Thus, some cells proliferate slowly while others proliferate faster, with most cells distributed about the mean on a log-linear plot. Prior to the discovery of the IL-2 molecule (2) and IL-2 receptors (IL-2R) (3), T cell populations were known to follow this rate-normal distribution, common to all living cells. Mathematical analysis of proliferating cell populations indicated that individual cell variability in cell cycle transit times was stochastic, depending upon a “hidden variable.” However, once the “hidden” molecular variables of T cell cycle proliferation had been identified (the IL-2 concentration, IL-2R density, affinity of the IL-2/IL-2R interaction, and the duration of the IL-2/IL-2R interaction), experiments could be preformed for the first time revealing that as long as these crucial characteristics of T cell cycle progression were known, then the variability of cell cycle transit times were entirely predictable and deterministic, not probabilistic or left to chance.

These experiments and approaches were possible because of painstaking attention to the creation of critical cell clones and homogeneous purified IL-2 molecules, as well as the development of the radiolabeled IL-2 binding assay, and monoclonal antibodies reactive with both IL-2 and its receptor. Moreover, the employment of the flow cytometer allowed us to proceed beyond studies of cell populations to quantify the intermolecular interactions of individual cells for the first time. Doreen Cantrell, a postdoctoral fellow skilled in flow cytometry, was critical to our experimental approach. Because the growth characteristics of all known cell populations are identical to those of T cell populations, it followed that individual cells of all other cell populations would demonstrate the same type of molecular determinants. Thus, the title of this article: “The interleukin-2 T cell system: A new cell growth model,” a new universal paradigm in cell and molecular biology (1).

The significance of these findings was obvious. As cells of all tissues, especially of metazoans, have identical growth characteristics of T cells, it followed that the cells of all metazoans are regulated in the same molecular fashion, i.e., cells are directed to undergo all-or-none (quantal) cell fate decisions by critical molecular concentrations. Furthermore, as all malignant cells arise from a single cell, so that all malignancies are clonal in origin, a gain of function mutation of one of the genes encoding the molecular determinants of cell cycle progression could obviate the strict cytokine/receptor/signaling pathways normally controlling the decision to divide, thereby resulting in a neoplastic cell growth (4). In other words, a critical “driver mutation” can put the cell on “autopilot.” From the viewpoint of the immune system, Burnet’s “Clonal Selection Theory,” derives from the observation that antigen-reactive clones of immune cells are selected by antigen, but subsequent to selection, each clone undergoes a proliferative clonal expansion (5). It is this clonal proliferative expansion that is determined by the molecular parameters discovered in this series of experiments focused on IL-2 and T cells. Accordingly, the regulatory cells and other cytokine molecules that influence the concentrations of the IL-2 molecules, their receptors, and the molecules of their signaling pathways ultimately determine the tempo, magnitude, and duration of immune responses. That is, internally derived hormones and their receptors regulate the immune system, just as every other bodily system is regulated, and immunity is not regulated solely by environmental antigens from without, a previous central dogma of immunology.

For immunologists, it is especially noteworthy that the lymphocyte antigen receptors function in an identical fashion to convert TCR signals to a digital control of cytokine gene expression (6–10).

These findings and considerations, led me to propose “The Quantal Theory of Immunity” (11–14). Thus, the immune system is regulated at the systemic (population) level by the number of clones responding and the extent of the proliferative expansion of each clone as originally stated by Burnet. However, at the level of individual cells, participation in an immune response is regulated in a quantal (all-or-none) fashion, and this quantal decision is determined by the absolute number of intermolecular interactions, which the cells somehow “count.” Once the crucial number has been surpassed, the cell responds in a quantal fashion. It goes without mentioning that all of the molecules and cells that participate in an immune response obey identical principles.

## Conflict of Interest Statement

The author declares that the research was conducted in the absence of any commercial or financial relationships that could be construed as a potential conflict of interest.
